# TCM nonpharmacological interventions for chronic low-back pain: A protocol for systematic review and network meta-analysis

**DOI:** 10.1097/MD.0000000000022547

**Published:** 2020-10-02

**Authors:** Haiyang Yu, Haiyan Wang, Tao Ma, Ailing Huang, Zengpeng Lu, Xiaogang Zhang

**Affiliations:** a Clinical College of Chinese Medicine, Gansu University of Chinese Medicine; b Department of Orthopedics; c Department of Acupuncture and Moxibustion, Affiliated Hospital of Gansu University of Chinese Medicine, Lanzhou, Gansu Province; d College of Acupuncture and Tuina, Chengdu University of Traditional Chinese Medicine, Chengdu, Sichuan Province, China.

**Keywords:** chronic low back pain, protocol, systematic review, TCM nonpharmacological interventions

## Abstract

**Background:**

chronic low back pain (CLBP) are common symptoms bothering people in daily life. Traditional Chinese medicine (TCM) nonpharmacological interventions are gaining an increasing popularity for CLBP. Nevertheless, the evidence of efficacy and safety of random controlled trials (RCTs) remains controversial. This study aims to evaluate the efficacy and acceptability of different TCM nonpharmacological therapies by systematic review and network meta-analysis.

**Methods:**

According to the strategy, The authors will retrieve a total of 7 electronic databases by September 2020, including PubMed, the Cochrane Library, EMbase, China National Knowledge Infrastructure, China Biological Medicine, Chongqing VIP, and Wan-fang databases After a series of screening, 2 researchers will use Aggregate Data Drug Information System and Stata software to analyze the data extracted from the randomized controlled trials of TCM nonpharmacological interventions for CLBP. The primary outcome will be the improvement of Pain intensity and functional status/disability and the secondary outcomes will include lobal improvement, health-related quality of life, satisfaction with treatment, and adverse events. Both classical meta-analysis and network meta-analysis will be implemented to investigate direct and indirect evidences on this topic. The quality of the evidence will be evaluated using the Grading of Recommendations Assessment, Development and Evaluation instrument.

**Results:**

This study will provide a reliable evidence for the selection of TCM nonpharmacological therapies in the treatment of CLBP.

**Conclusion:**

This study will generate evidence for different TCM nonpharmacological therapies for CLBP and provide a decision-making reference for clinical research.

**Ethics and dissemination:**

This study does not require ethical approval. The results will be disseminated through a peer-reviewed publication.

**OSF registration number:**

DOI 10.17605/OSF.IO/4H3Y9.

## 1 Introduction

Low back pain (LBP) is a very common disorder, and it causes significant pain, impairment of daily activities, and decreases quality of life.[^1^,^2^,^3^] With approximately 84% of adults experiencing an episode of LBP at some point during their lifetime and variable recurrence rates (5%–60%).^[4]^ According to 2017 global statistics, low back pain accounts for 7.3% point prevalence of activity-limiting illnesses, affecting 54 billion people at all ages.^[5]^ It is associated with more global disability than any other condition. Chronic low back pain (CLBP) refers to LBP has persisted for more than 3 months.[^1^,^2^] CLBP carries an enormous economic burden from both direct (e.g., treatment) and indirect (e.g., lost work productivity) costs.^[6]^ In the United States, approximately $100 billion of medical expenses is spent annually for back pain with an additional $50 billion arising from indirect costs due to the lost in productivity and disability benefit payments.^[7]^ CLBP is also associated with impaired quality of life, mobility and daily function as well as social isolation, disability, and depression.[^8^,^9^] It is a major health problem leads to more years living with disability than any other musculoskeletal condition.^[7]^ Although financial burden and disability attributed to chronic low back pain is substantially different between countries, the incremental impact of the worldwide health care system is expected to be significant in the coming decades.^[8]^

Even though conventional treatments such as medication or surgery have shown some efficacy against lower back pain,[^10^,^11^] these treatments were not always effective, and even had some serious adverse effects including diarrhea, nausea, vomiting, muscle spasms or leg cramps, insomnia, headache, and abnormal dreams. Consequently, many individuals have turned their attention to some other treatments, such as complementary and alternative medicine (CAM). Traditional Chinese medicine (TCM), as a main component of CAM based on current knowledge, has been widely applied in management of chronic conditions including CLBP in the world.

Recently, an increasing amount of evidenced-based medicine (EBM) evidences have revealed that TCM nonpharmacological therapies including acupuncture, acupressure, cupping, moxibustion, tuina, and tai chi, have potentially positive effects in CLBP management.

Acupuncture plays an important role in TCM treatment of pain.[^12^,^13^] Recently, clinical studies have proved acupuncture is beneficial for the treatment of CLBP.[^14^,^15^,^16^,^17^,^18^,^19^,^20^,^21^] Meanwhile, moxibustion is a form of TCM that has been widely used in East Asia for thousands of years.^[22]^ In recent years, a number of basic and clinical studies have proved moxibustion is beneficial for the treatment of CLBP.[^23^,^24^,^25^,^26^,^27^] Acupressure, another treatment modality of Chinese traditional medicine, is a gentle but firm pressing stimulation on meridians and acupoints, and the efficacy of acupressure in pain relief associated with LBP has been proven by several clinical randomized controlled trials.[^28^,^29^,^30^,^31^,^32^,^33^] Tuina, a manual therapy in traditional Chinese medicine, emphasizes anatomy and physiology when used for neuromusculoskeletal disorders, which is currently widely used for the treatment of CLBP.[^34^,^35^,^36^,^37^] And Tai Chi, a mind-body exercise therapy, is typically used to demonstrate positive effects on CLBP.[^38^,^39^]

Yet no comparative effectiveness investigation was done, which may exert some influence on clinical decision-making and the implemention of health economic policies. Due to the complex components, we aim to examine comparative effectiveness of TCM nonpharmacological interventions in CLBP by conducting a systematic review and network meta-analysis.

## 2 Methods

### 2.1 Protocol and registration

This protocol follows the Preferred Reporting Items for Systematic Reviews and Meta-Analyses Protocols (PRISMA-P) guidelines.^[40]^ The NMA protocol has been registered on Open Science Framework (OSF) platform (https://osf.io/g9rux/), registration number: DOI 10.17605/OSF.IO/4H3Y9

### 2.2 Eligibility criteria

#### 2.2.1 Type of participant

Studies of adult (>18 years old) patients with chronic low back pain which lasted for more than 12 weeks were eligible, regardless of pain cause, intensity, and radiation pattern. There will be no restriction on sex, ethnicity, disease duration or disease severity.

#### 2.2.2 Type of interventions and comparators

Interventions in the treatment group will include any kinds of TCM non-pharmacological interventions for CLBP, including acupuncture, acupressure, cupping, moxibustion, tuina, tai chi, etc. We also include TCM non-pharmacological interventions in combination with other conservative treatments. However, combined interventions consisting of 3 or more therapies or with potential safety problems will be excluded. Control interventions will include no treatment, sham acupuncture and sham moxibustion, waiting list, oral drugs, any active treatment. Studies comparing the same kind of TCM nonpharmacological interventions, but with different sessions, acupoints selections will be taken as the identical node in network analysis.

#### 2.2.3 Type of outcomes

##### 2.2.3.1 Primary outcomes

The primary outcome of the study is the Pain intensity and functional status/disability, as measured by validated assessment tools. The assessment tools include pain intensity, including Visual Analogue Scale (VAS),^[41]^ Numerical Rating Scale (NRS)^[42]^), and disability (on Roland Morris Disability Questionnaire (RMDQ),^[43]^ Oswestry Disability Index (ODI)^[44]^).

##### 2.2.3.2 Secondary outcomes

The secondary outcomes will include the following: global improvement using validated tools such as the Japanese Orthopedic Association (JOA) score,^[45]^ health-related quality of life using validated tools such as the Short Form Survey Instrument (SF-36),^[46]^ satisfaction with treatment, and adverse events.

#### 2.2.4 Study design

This study is a systematic review and network meta-analysis of RCTs with TCM non-pharmacological therapies on CLBP. This research will include all relevant RCTs using TCM non-pharmacological therapies for CLBP and the first period in randomized cross-over trials, regardless of publication status. Quasi-RCTs, review documents, clinical experience, and case reports. Moreover, we will only search English and Chinese literature in this study. And we will remove the studies without comparable baselines and duplicate publications.

### 2.3 Literature retrieval strategy

Computer retrieval of published RCTs of Traditional Chinese medicine nonpharmacological interventions for CLBP is conducted in PubMed, the Cochrane Library (issue 9, 2020), EMbase, China National Knowledge Infrastructure (CNKI), China Biological Medicine (CBM), Chongqing VIP, and Wan-fang databases. The time limit of document retrieval is from the establishment of each database to September 30, 2020. Using medical subject heading (MeSH) terms and key words to identify RCTs with the limitation of Chinese and English language. In addition, inclusive literature from the field and references from previous evaluations will be manually retrieved to find other potentially relevant articles. Chinese search terms mainly include: “chronic low-back pain.”; English search words include “chronic low-back pain.”, “CLBP”, “acupuncture”, “moxibustion”, “cupping”, “tui na”“Tai Chi”, etc. Taking PubMed as an example, the initial retrieval strategy is shown in Table 1 and will be adjusted according to the specific database.

**Table 1:**
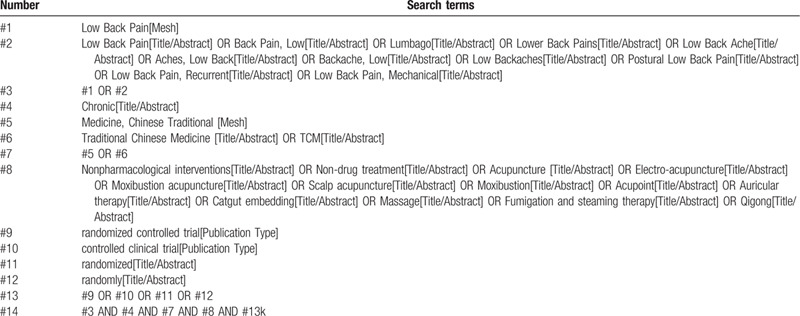
Search strategy of the PubMed.

### 2.4 Literature selection and data extraction

**Figure 1:**
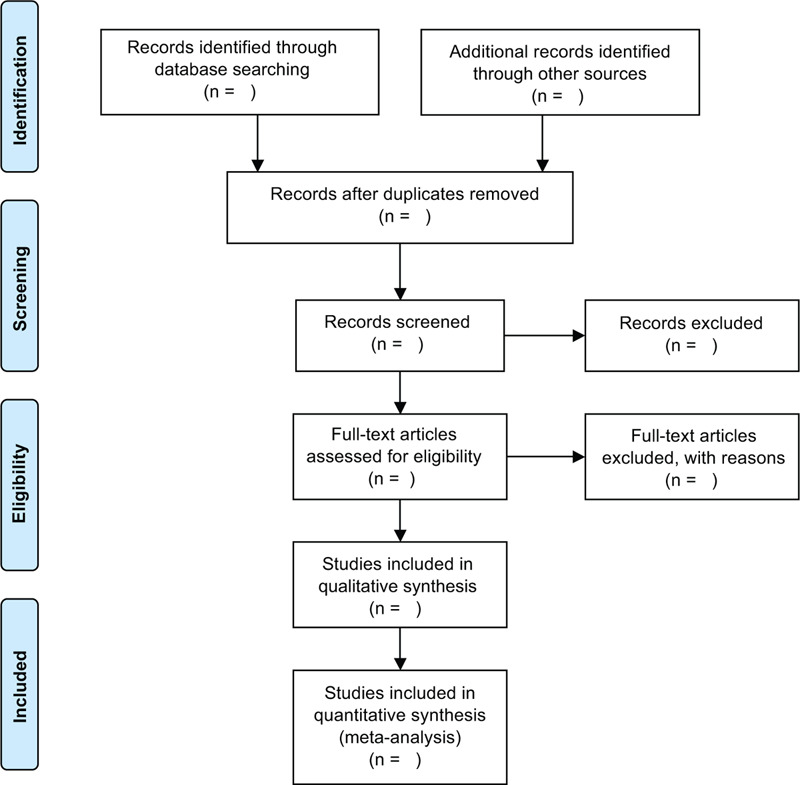
Flow chart of literature screening.

The study selection program will follow the Prisma guidelines, As shown in Figure 1, Haiyang Yu and Haiyan Wang will independently screen literatures according to inclusion and exclusion criteria and cross-checked against:

1. Preliminary screening of the literature through Endnote software to remove duplicates;
2. By reading the title and preliminaryly screening the abstract, exclude the literature that obviously does not meet the inclusion criteria;
3. Download and read the full text for re-screening.

At the end of the filtering, the extracted features are recorded using a pre-designed data table. These features include title, journal, author, publication year, country, sample size, gender, mean age, intervention, comparator, course of treatment, outcome measures, and follow-up time. If there is any disagreement, the third researcher Tao Ma will be asked to assist in the judgment. At the same time, the key factors of bias risk assessment are extracted.

### 2.5 Quality assessment

The quality of systematic review reflects the risk of bias or validity in its process and results, as well as the reliability of the included studies. The quality of the included studies will be assessed according to the Cochrane Reviewers’ Handbook. Two trained researchers Haiyang Yu and Tao Ma will independently evaluate the risk of bias of the included studies. If the results are disputed, they will be submitted to the corresponding author (Xiaogang Zhang) of this study for review and determination.

Cochrane Reviewers’ Handbook will be used to assess the risk of RCTs being included in NMA, including^[47]^:

1. random sequence generation;
2. allocation concealment;
3. blinding of the subjects and researchers;
4. blinding of outcome assessment;
5. incomplete outcome data;
6. selective reporting;
7. other bias.

### 2.6 Data synthesis and statistical methods

#### 2.6.1 Network meta-analysis

This study uses ADDIS 1.16.8 based on Bayesian framework for NMA.^[48]^ Odds ratios (ORs) or standardized mean differences (SMD) will be modeled using Markov chain Monte Carlo methods, both with 95% confidence intervals (CIs). Preset model parameters: 4 chains are used for simulation analysis, with an initial value of 2.5, a step size of 10, 20,000 annealing times, and 50,000 simulation iterations. The network evidence plot will be generated according to different outcome. According to the results of the NMA, rank probability plot of various TCM nonpharmacological therapies will be generated and sorted by dominance, with Rank1 being the optimal sort.

#### 2.6.2 Consistency assessments /statistical model selection

The Node-split model is used to check for consistency between direct and indirect evidence. If there is no statistical difference (*P* > .05) between direct comparison and indirect comparison, the consistency model is used, whereas the inconsistency model is used for analysis. If the consistency model is adopted, then the stability of the results is verified by the inconsistency model: when the inconsistency factors including 0, at the same time inconsistency standard deviation including 1 says the result of consistency model is more stable and reliable. At the same time, various analysis models are iterated with preset parameters, and the convergence of iteration effect is judged by potential scale reduced factor (PSRF). When the PSRF value is close to or equal to 1 (1≤PSRF≤1.05), the convergence is complete, the model has good stability, and the conclusion of analysis is reliable. If the PSRF value is not in this range, the iteration continues manually until the PSRF value reaches the range standard.

#### 2.6.3 Heterogeneity test

Before the combination of effect size, we will use Stata to assess available study and patient characteristics to ensure similarity and to investigate the potential effect of heterogeneity on effect estimates. When inter-study heterogeneity exists, the random effect model is used. For comparison of each pair, heterogeneity is assessed by the statistic *I* ^2^ value. When *I* ^2^ > 50%, it indicates that there is heterogeneity between studies, and the source of heterogeneity should be further searched. When *I* ^2^ < 50%, inter-study heterogeneity is considered to be small or there is no obvious heterogeneity.

#### 2.6.4 Sensitivity analysis

If necessary, the sensitivity analysis will be used to assess the effect of each study on the random effects model. The sensitivity of the general combined effect of all outcome indicators is analyzed by the exclusion method. That is, each study is excluded, and the remaining studies will be re-analyzed to identify the stability of the results. If there is no qualitative change in the combined effect showed in the results, the results are stable.

#### 2.6.5 Subgroup analysis

If necessary, we will conduct a subgroup analysis of duration of treatment, age, the course of CLBP, and research quality.

#### 2.6.6 Small sample effect/publication bias

If 10 or more studies are included in the NMA, a comparison-adjusted funnel plot is developed using Stata to evaluate the presence of small sample effects or publication bias in the intervention network. Descriptive analysis will be carried out through the symmetry of funnel plot. If the plot is asymmetric and there is no inverted funnel shape, it indicates that there may be publication bias. This may be related to the difficulty in the publication of the literature with negative results and the low quality of the inclusion methods.

#### 2.6.7 Dealing with missing data

If the required data is lost or incomplete, we will contact the corresponding author of the original document or the relevant email address of the first author. If there is no response, the record is excluded.

#### 2.6.8 Evaluating the quality of the evidence

To grade evidence quality and understand the current situation of evidence rating thereby analyzing possible problems, The Grading of Recommendations Assessment, Development and Evaluation (GRADE) instrument will be used to assess the quality of evidence in the NMA.^[49]^ Based on the risk of bias, inconsistency, imprecision, indirection, and publication bias, GRADE grades evidence quality into 4 levels: high, medium, low, and very low.

## 3 Discussions

As a consequence of the acceleration of global aging, sedentary lifestyle increasing and the gain of average weight, CLBP has become a global condition with high incidence.^[50]^ More than 70% of people suffer from lower back pain in developed countries.^[51]^ CLBP accounts for financial burden and disability.[^6^,^7^,^8^] Therefore, effective and safe treatment is especially crucial in overcoming low back pain and disability related to the chronic condition.

With an increasing amount of publications on nonpharmacological interventions for patients with CLBP in recent years, we would like to figure out which has the relatively optimal effect and safety among those interventions. Given that systematic reviews with good quality can help provide best evidence in clinical practice, and a network meta-analysis can offer a ranking result based on comparative effectiveness, safety and costs, we conceive and design this study protocol. We will assess the quality of evidence with the GRADE framework: risk of bias, heterogeneity or inconsistency, imprecision, indirectness, and publication bias. Our study will generate evidence of TCM in the treatment of CLBP and help to reduce the uncertainty about the effectiveness of CLBP management.

This study has a number of limitations. First, a number of studies we included were of low quality. Few RCTs comparing interventions and controls were available, limiting the number of studies that could be included in the meta-analysis. Second, a few included reports were therapies which cannot be blinded to participants, especially acupuncture. However, blinding of outcome assessment and single-blind methodologies should be used where possible to reduce the potential for any biases. Third, since there were very few trails had long-term follow-up, it is impossible to analyze the long-term effect. Last, the review may be susceptible to publication bias, though this was not evident when funnel plots were examined. As reported, data were markedly heterogeneous with a significant amount of unreported data.

## Author contributions

All the authors have approved the publication of the protocol.

**Conceptualization:** Haiyang Yu, Haiyan Wang, Tao Ma.

**Data curation:** Haiyang Yu, Haiyan Wang, Ailing Huang.

**Formal analysis:** Zengpeng Lu.

**Funding acquisition:** Xiaogang Zhang.

**Methodology:** Haiyang Yu, Haiyan Wang, Tao Ma.

**Project administration:** Haiyan Wang, Ailing Huang.

**Writing – original draft:** Haiyang Yu, Haiyan Wang.

**Writing – review & editing:** Xiaogang Zhang.
